# The Arbuscular Mycorrhizal Fungus *Glomus viscosum* Improves the Tolerance to Verticillium Wilt in Artichoke by Modulating the Antioxidant Defense Systems

**DOI:** 10.3390/cells10081944

**Published:** 2021-07-30

**Authors:** Alessandra Villani, Franca Tommasi, Costantino Paciolla

**Affiliations:** Department of Biology, University of Bari “Aldo Moro”, Via E. Orabona 4, 70125 Bari, Italy; alessandra.villani@uniba.it (A.V.); franca.tommasi@uniba.it (F.T.)

**Keywords:** Verticillium wilt, *Glomus viscosum* Nicolson, arbuscular mycorrhizal fungi, oxidative stress, antioxidant systems, defense ability

## Abstract

Verticillium wilt, caused by the fungal pathogen *Verticillium dahliae*, is the most severe disease that threatens artichoke (*Cynara scolymus* L.) plants. Arbuscular mycorrhizal fungi (AMF) may represent a useful biological control strategy against this pathogen attack, replacing chemical compounds that, up to now, have been not very effective. In this study, we evaluated the effect of the AMF *Glomus viscosum* Nicolson in enhancing the plant tolerance towards the pathogen *V. dahliae*. The role of the ascorbate-glutathione (ASC-GSH) cycle and other antioxidant systems involved in the complex network of the pathogen-fungi-plant interaction have been investigated. The results obtained showed that the AMF *G. viscosum* is able to enhance the defense antioxidant systems in artichoke plants affected by *V. dahliae*, alleviating the oxidative stress symptoms. AMF-inoculated plants exhibited significant increases in ascorbate peroxidase (APX), monodehydroascorbate reductase (MDHAR), and superoxide dismutase (SOD) activities, a higher content of ascorbate (ASC) and glutathione (GSH), and a decrease in the levels of lipid peroxidation and hydrogen peroxide (H_2_O_2_). Hence, *G. viscosum* may represent an effective strategy for mitigating *V. dahliae* pathogenicity in artichokes, enhancing the plant defense systems, and improving the nutritional values and benefit to human health.

## 1. Introduction

The artichoke (*Cynara scolymus* L.) is a horticultural species of relevant economic interest belonging to the Asteraceae family, widely cultivated in the Mediterranean basin and widespread throughout the world [1,2]. This perennial crop is well known for the antioxidative, antimicrobial, and probiotic properties of its edible parts, including the inner fleshy leaves (bracts) and the receptacle [1,3]. Several studies have demonstrated that even some non-food by-products of artichokes, including leaves, external bracts, and stems, exhibit beneficial and therapeutic effects and are widely used as hepatoprotective [4], antioxidant [5,6], anticarcinogenic [7], hypoglycemic [8], and hypocholesterolemic [9] agents. The health-promoting properties and important nutritional values of artichokes have been extensively related to inulin, fibers, and minerals, and to the high content of some bioactive phenolic compounds, such as caffeoylquinic acid derivatives and flavonoids, showing a strong scavenging activity against reactive oxygen species (ROS) and free radicals [1,2,10].

As the artichoke is an herbaceous plant which survives in the field for several years, a large number of insects, nematodes, bacteria, fungi, and viruses can attack and invade its seeds, roots, foliage, and vascular system, causing numerous diseases [11,12]. Verticillium wilt, caused by the fungus *V. dahliae* Kleb., represents one of the greatest threats to the artichoke plantation worldwide [13,14,15]. This soil-borne pathogen is distributed throughout the world, and it affects over 400 plant species that belong to 14 plant families, showing a broad range of symptoms and causing significant yield losses and quality reduction for most of the host plant species [15,16]. Among these species, almond, apricot, artichoke, cabbage, cauliflower, chrysanthemum, cotton, cucurbits, olive, peach, potato, strawberry, sunflower, and tomato were defined as the most severely affected host crops [16]. *V. dahliae* causes a monocyclic disease divided into three phases: dormant, parasitic, and saprophytic [17,18]. In the presence of a host, the disease cycle begins with germination of microsclerotia that are released in the soil with the decomposition of plant materials where they can remain viable for up to 14 years. Hyphae from germinating microsclerotia can colonize and penetrate the roots of host plants, following a slow progression through the vascular (xylem) tissues, where conidia can grow and continue the colonization, leading to xylem malfunctioning and reduced movement of water and nutrients from the roots to the foliage of the infected host [17]. Over the years, several strategies have been tested to manage diseases caused by *V. dahliae,* including selection of planting site, crop rotation and manipulation of fertility and irrigation, use of healthy planting material, selection of available resistant cultivars, fungicides treatments, and soil fumigants [15,18,19,20,21].

Methyl bromide has been widely used for decades as a soil fumigant for controlling Verticillium wilt until its complete phase-out in 2005 according to the Regulation EC 2037/2000 because of its threat to the environment, being one of the major ozone depleting substances, and to humans, causing lung injury and neurological effects. Some other fumigants have been tested, such as the mixture of 1,3-dichloropropene and chloropicrin, dazomet, and metam sodium, but are not very effective [22]. Similarly, several fungicides applied as foliar sprays, soil drenches, or granular preparations have been tested, but the effectiveness was observed only with high dosages, which many a times cause phytotoxic effects [23]. Therefore, the inability of fungicide and soil fumigants treatments to control the disease successfully, the unavailability of artichoke resistant cultivars as well as the inaccessibility of *V. dahliae* during infection and its long-term persistence in the field have required alternative strategies. Furthermore, the public concern over the environment pollution, ecosystem’s biodiversity, and food safety has enhanced research efforts towards eco-friendly practices for a sustainable agricultural management. To ensure that aim, beneficial microorganisms, such as AMF, could play a crucial role. AMF are symbionts, mainly belonging to the phylum Glomeromycota that form arbuscular mycorrhizal associations with the roots of over 80% of all vascular plants [24]. Numerous studies have demonstrated that AMF can improve water and mineral nutrient uptake from the soil by increasing the plant root surface area [25]. Initial stages of AMF colonization trigger an intracellular ROS burst in the host plant; however, this effect is transient and is overcome by enhanced activities of antioxidant enzymes [26]. Indeed, AMF increase the accumulation of secondary metabolites in several plants, including phenolic compounds, vitamins, and sugars [10,27], mitigate the oxidative burst of plants under abiotic stresses by increasing the activity of some antioxidants that are involved in the alleviation of oxidative damage caused by ROS [28,29,30], and protect host plants from pathogens, overcoming the harmful effects of abiotic and biotic stresses [31]. In particular, the efficacy of AMF in reducing the disease severity of Verticillium wilt has been demonstrated on olive [32,33], eggplant and tomato [34,35], pepper [35], oilseed rape (*Brassica napus* L. cv. Licosmos), and strawberry (*Fragaria ananassa* cv. Elsanta) [36] plants. Moreover, the presence of an autochthonous mycorrhizal consortium “Rhizolive consortium” on the early oxidative events induced in olive plants after *V. dahliae* inoculation stimulated the activity of antioxidant enzymes, reducing oxidative damage [37], and treatments with six AMF in two artichoke cultivars increased the level of total phenols and total antioxidant activity [20].

The increase in ROS is a common biochemical response to abiotic and biotic stresses in plants. Higher ROS levels in the cell could cause oxidative damage to DNA, lipids, and proteins. It is well known that the ROS level in cells is under the control of antioxidant systems, such as the ASC-GSH cycle [38], and enzymes, including SOD, catalase (CAT), and generic peroxidases (PODs), which have a pivotal role in defense mechanisms. The activity of those enzymes, with that of APX, a component of ASC-GSH cycle, is crucial for determining the steady-state level of superoxide anion and H_2_O_2_ in plant cells [39]. SOD dismutates superoxide anion to H_2_O_2_, which can be converted into oxygen and H_2_O by CAT, PODs, or APX. In the ASC-GSH cycle, the APX uses two molecules of ASC to reduce H_2_O_2_ to water, with the concomitant generation of two molecules of monodehydroascorbate (MDHA) [40]. MDHA is a radical with a short lifetime that is rapidly reduced to ASC by MDHAR, which is a flavin enzyme that utilizes NAD(P)H as electron donors. Dehydroascorbate (DHA), the oxidized form of ASC, can be reduced back to ASC by DHA reductase (DHAR), which utilizes GSH as an electron donor, leading to the formation of glutathione disulphide (GSSG), which is in turn re-reduced to GSH by NADPH, a reaction catalyzed by the glutathione reductase (GR).

The objective of this study was to test the ability of the AMF *G. viscosum* to moderate the metabolic alterations related to oxidative stress in artichoke plants attacked by *V. dahliae*. In particular, a deeper investigation of the involvement of the defense systems in the fungi-plant interaction, by evaluating the level of the components of the ASC-GSH cycle and the antioxidant enzymes (CAT and SOD), led to a better understanding of the biochemical mechanism on the basis of this complex network among the plant, pathogen, and AMF.

## 2. Materials and Methods

### 2.1. Chemicals

All reagents used in this study were of the highest grade available, purchased from Sigma–Aldrich (Milan, Italy) and used without further purification. Ultrapure water was produced by a Milli-Q system 84 (Millipore, Bedford, MA, USA).

### 2.2. Plant Material and Sampling

The material analyzed was obtained from the experimental farm of “P. Martucci” of the University of Bari in Valenzano, Apulia, Italy. Plants of artichokes (*Cynara cardunculus* L. var. *scolymus* L. cv. Violetto di Provenza), obtained by micropropagation, were transplanted in pots containing a commercial peat mixture soil enriched with nutrients (organic carbon 46%, organic nitrogen 1–2%, organic matter 80%) and mixed with perlite at a 2:1 (*v*/*v*) ratio. The peat mixture was sterilized and used to fill 10 cm diameter pots. Prior to transplantation, half of the microplants were inoculated with 10 g of crude inoculum of the AMF *G. viscosum*, as described by Morone Fortunato et al. [41]. Non-mycorrhizal plants were used as controls. Acclimatization took place in greenhouse conditions at 18 ± 2 °C with mist and a relative humidity level reduced from 85–90% to 55–60% over 20 days. After 60 days, 48 non-mycorrhizal plants and 48 inoculated plants were transplanted to the open field, according to a randomized block design with treatments replicated three times. Each block consisted of eight plants, and the spacing used was 1.2 m between rows and 1 m between plants for all the treatments. A portion of the field was inoculated 15 days before the transplantation with an inert substrate enriched with mycelium, microsclerotia, and spores of *V. dahliae* isolated from naturally infected artichokes. Then, the treatments were established as follows: (i) non-mycorrhizal plants (Ctrl), (ii) non-mycorrhizal plants inoculated with *V. dahliae* (I), (iii) mycorrhizal plants (M), (iv) mycorrhizal plants inoculated with pathogen (MI).

### 2.3. Source of V. dahliae Isolates

Isolates of *V. dahliae* were recovered from symptomatic artichoke plants in a field with a known history of Verticillium wilt. Segments of 5 mm long surface-sterilized stems from infected host plants were transferred on a potato dextrose agar (PDA), (Difco, Detroit, MI, USA) supplemented with streptomycin sulphate (100 ppm) and incubated at 27 °C for 10 days in darkness. Colonies of *V. dahliae* were morphologically identified visually and microscopically, subcultured on PDA without antibiotics, and then mixed with vermiculite for the inoculation in the field. A further identity confirmation was provided by sequencing. PCR amplification and sequencing were performed using the ribosomal internal transcribed spacer region (*ITS*) as the locus, according to Inderbitzin et al. 2011 [42]. Species identification was confirmed by BLASTn against the NCBI GenBank database (http://www.ncbi.nlm.nih.gov, accessed on 14 January 2021).

### 2.4. Disease Assessments

A scoring metric was used to assess disease severity of the artichoke plants over time. Wilt severity was rated according to Uppal et al.’s [43] scoring system as follows: 0, no wilt symptoms; 1, inter-veinal chlorosis on the lower leaves; 2, moderate necrosis and defoliation of the lower leaves; 3, severe leaf necrosis and defoliation; and 4, severe defoliation accompanied by pronounced stunting, chlorosis, and necrosis of the remaining leaves. Furthermore, a rating scale was also established to evaluate the severity of vascular browning of artichoke stems. This scale consisted of the following grades: 0, no vascular browning; 1, trace to less than 9% of the stem cross-section showing a vascular browning; 2, 10–24% of the stem cross-section with a vascular browning; 3, 25–49% of the stem cross-section showing vascular browning; and 4, over 50% of the stem cross-section exhibiting vascular browning. In addition, the effect of mycorrhization on the growth of artichoke plants was assessed by counting the number of flower heads per plant. Data were analyzed with analysis of variance (ANOVA), and the means were compared by the Duncan test.

### 2.5. Determination of ASC and GSH Pool Contents

Foliar tissues (20 g) were homogenated at 4 °C with three volumes of 5% (*w/v*) metaphosphoric acid and then centrifuged for 15 min at 20,000× *g*. The resulting supernatant was used for analysis of the ASC and GSH pool content according to Zhang and Kirkham [44]. 

### 2.6. Enzyme Assays

For determination of antioxidant enzyme activities, samples were homogenized according to Mastropasqua et al. [45] with slight modifications. Briefly, twenty grams of foliar tissues were homogenized in 50 mM Tris–HCl, pH 7.8 containing 0.3 mM mannitol, 1 mM EDTA, 10 mM MgCl_2_, 1% (*w/v*) polyvinyl-pyrrolidone (PVP), and 0.05% (*w/v*) cysteine 1%, at 4 °C. The homogenate was filtered through four layers of cheesecloth and centrifuged (20,000× *g*, 20 min, 4 °C). The supernatant was desalted by dialysis against 50 mM Tris-HCl, pH 7.8, and used for spectrophotometric analysis of the total proteins and enzymatic activities.

The total protein content of samples was measured with a Protein Assay kit from Bio-Rad (Hercules, CA, USA) with bovine serum albumin as the standard [46]. The reproducibility of the Bio-Rad kit, expressed as the coefficient of variation (%CV), is 2% approximately; the lower limit of the detection for protein molecular weight is 3000 to 5000 Daltons.

The enzymatic spectrophotometric assays for the determination of cytosolic APX (EC 1.11.1.11), DHAR (EC 1.8.5.1), CAT (EC 1.11.1.6), GR (EC 1.8.1.7), MDHAR (EC 1.6.5.4), and SOD (EC 1.15.11) were performed according to Paciolla et al. [47] and Mastropasqua et al. [48].

### 2.7. Electrophoretic Analyses

Native-Polyacrilamide Gel Electrophoresis (Native-PAGE) was performed on PAGE (4.3% T; 7.3% C) with a running buffer composed of 4 mM Tris-HCl pH 8.3 and 38 mM glycine. In each lane of the gel, 200 μg of total proteins were loaded. After the electrophoretic run, the gels were washed with distilled water and incubated in specific buffers for the detection of APX and CAT, as described in Paciolla et al. [49]. For the SOD, the activity on the gel was visualized by incubating it in 0.053 Tris–HCl buffer pH 8.2 containing 0.21 mM riboflavin and 0.244 mM nitro-blue tetrazolium (NBT) in the dark; after 15 min, achromatic bands on a grey background appeared, a 50% glycerol solution was used to block the reaction.

For densitometric analysis of SOD activity, the gel was acquired utilizing the Gel/ChemiDoc and Quantity One software (BioRad Laboratories Inc., Milan, Italy) to obtain information on the changes in the activity of each band due to different treatments. A relative value of 100 was assigned to the intensity of the bands of Ctrl and I samples.

### 2.8. Lipid Peroxidation Analysis and H_2_O_2_ Content 

For lipid peroxidation, plant material was ground with four volumes of 0.1% (*w/v*) trichloroacetic acid (TCA). The homogenate was centrifuged at 12,000 × *g*, for 10 min, at 4 °C. One mL of the supernatant was mixed with 4 mL of 20% TCA containing 0.5% (*w/v*) thiobarbituric acid (TBA). The level of cell lipid peroxidation was evaluated in terms of malondialdehyde (MDA) content determined by the TBA reaction as described by Zhang and Kirkham [44]. Intracellular H_2_O_2_ concentration was evaluated according to Lee and Lee [50].

### 2.9. Statistical Analysis

The biochemical data presented are the means of five different experiments. Statistical analysis was done using Student’s *t*-test, with level of significance for *p* < 0.05 and highly significant for *p* < 0.01; the standard deviation (SD) was calculated, and its range is shown in the figures. Data presented for disease assessments are the average of three experiments with three replicates and were analyzed with ANOVA with *p* ≤ 0.05; the means were compared by the Duncan test. 

## 3. Results

### 3.1. Disease Assessments 

The results showed a beneficial effect of mycorrhization in containing artichoke wilt. In particular, the AMF *G. viscosum* significantly reduced the disease severity, measured by symptoms’ development on leaves, on the MI treatment, while it slightly reduced the vascular browning (Table 1). In addition, the beneficial effect of mycorrhization on productivity was observed.

### 3.2. Ascorbate and Glutathione Pool Content

**Figure 1:**
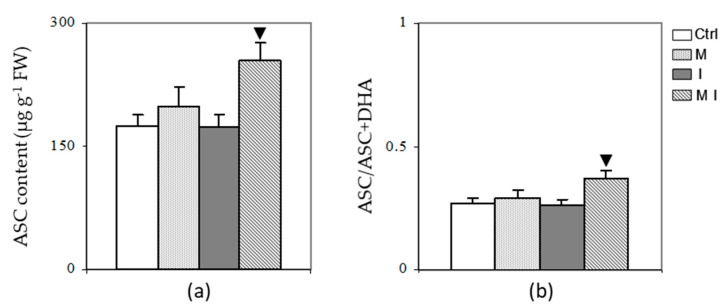
Ascorbate (ASC) content (**a**) and ASC redox ratio (**b**) in artichoke control plants (Ctrl), in mycorrhizal plants (M), in plants inoculated with *Verticillium dahliae* (I), and in mycorrhizal plants inoculated with *V. dahliae* (MI). The results are given as the mean values of at least five experiments ± SD; ▼ indicates values significantly different from the artichoke inoculated with *V. dahliae* (I) by the Student’s *t* test with *p* < 0.05. FW, fresh weight.

The mycorrhizal plants inoculated with the pathogen *V. dahliae* (MI) showed an increase (*p* < 0.05) in ASC content as compared to non-mycorrhizal inoculated plants (I), while no significant increment was observed in mycorrhizal plants (M) with respect to non-mycorrhizal plants (Ctrl) (Figure 1, Panel a). DHA did not differ significantly among the treatments (data not shown), and, hence, the ascorbate redox ratio (ASC/ASC + DHA) was higher in MI than in the other treatments (Figure 1, Panel b).

**Figure 2:**
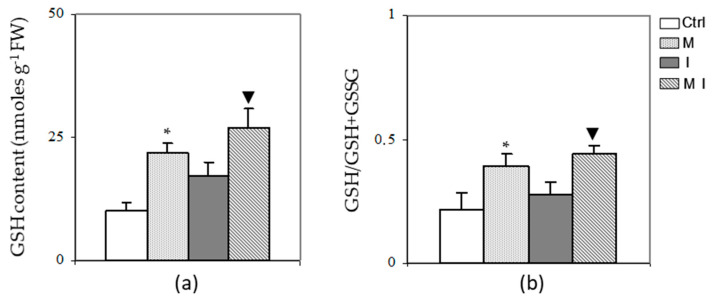
**Glutathione** (GSH) content (**a**) and glutathione redox ratio (**b**) in artichoke control plants (Ctrl), in mycorrhizal plants (M), in plants inoculated with *Verticillium dahliae* (I), and in mycorrhizal plants inoculated with *V. dahliae* (MI). The results are given as the mean values of at least five experiments ± SD; * indicates values significantly different from the control (Ctrl) by the Student’s *t* test with *p* < 0.05; ▼ indicates values significantly different from the artichoke inoculated with *V. dahliae* (I) by the Student’s *t* test with *p* < 0.05. FW, fresh weight.

In both mycorrhizal plants (M and MI), an increase (*p* < 0.05) in GSH content was observed (Figure 2, Panel a) with respect to the control and the inoculated plants (I), respectively. In addition, the GSSG content was similar in all samples (data not shown), therefore, the glutathione redox ratio (GSH/GSH + GSSG) was higher in M and MI with respect to the control and the plants inoculated with the pathogen (I), respectively (Figure 2, Panel b).

### 3.3. Antioxidant Enzyme Assays

**Figure 3:**
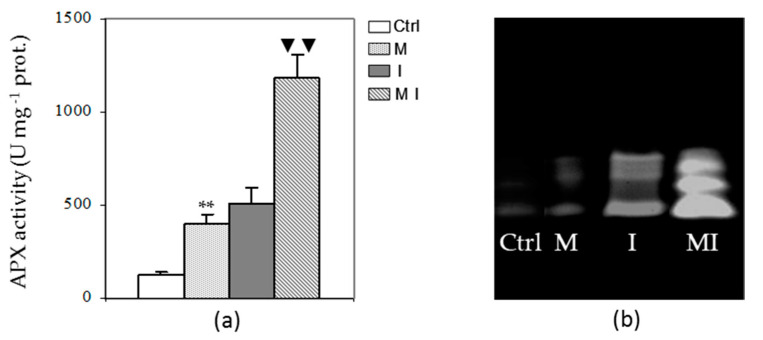
Spectrophotometric activity (**a**) and electrophoretic profile after Native-PAGE (**b**) of ascorbate peroxidase (APX) in the cytosolic fraction of artichoke control plants (Ctrl), mycorrhizal plants (M), plants inoculated with *Verticillium dahliae* (I), and in mycorrhizal plants inoculated with *V. dahliae* (MI); 1 U = 1 nmol of ascorbate oxidized min^−1^; prot. = proteins. The results are given as the mean values of at least five experiments ± SD; * * and ▼▼ indicate values significantly different from the control (Ctrl) and from the artichoke inoculated with *V. dahliae*, respectively, by the Student’s *t* test with *p* < 0.01.

Activities of the enzymes in the ascorbate–glutathione cycle, including APX, DHAR, MDHAR, and GR, showed difference trends. The activity of APX was significantly higher in MI compared to I as shown in Figure 3 (Panel a). Similarly, M showed higher APX activity with respect to the control. These results were confirmed by Native-PAGE, which showed that enzyme activity of APX was higher in M and MI than in Ctrl and I, respectively. Furthermore, the native electrophoretic pattern of APX showed a total number of three isoforms with same migration rate in all samples (Figure 3, Panel b).

**Figure 4:**
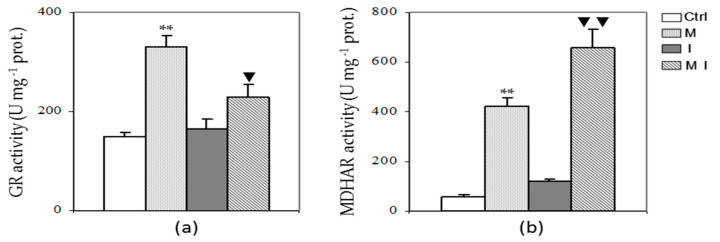
Enzymatic activity of glutathione reductase (GR) (**a**) and monodehydroascorbate reductase (MDHAR) (**b**) in the cytosolic fraction of artichoke control plants (Ctrl), mycorrhizal plants (M), plants inoculated with *Verticillium dahliae* (I), and in mycorrhizal plants inoculated with *V. dahliae* (MI). For GR activity, 1 U = 1 nmol of NADPH oxidized min^−1^; for MDHAR activity, 1 U = 1 nmol of NADH oxidized min^−1^; prot. = proteins. The results are given as the mean values of at least five experiments ± SD; * * indicates values significantly different from the control (Ctrl) by the Student’s *t* test with *p* < 0.01; ▼ and ▼▼ indicate values significantly different from the artichoke inoculated with *V. dahliae* (I) by the Student’s *t* test with *p* < 0.05 and 0.01, respectively.

The assay of GR and MDHAR activities revealed a trend similar to APX, showing both in M and MI a significant increment compared to Ctrl and I plants, respectively (Figure 4, Panels a and b). In contrast, the activity of DHAR remained almost unchanged among all treatments (data not shown).

**Figure 5:**
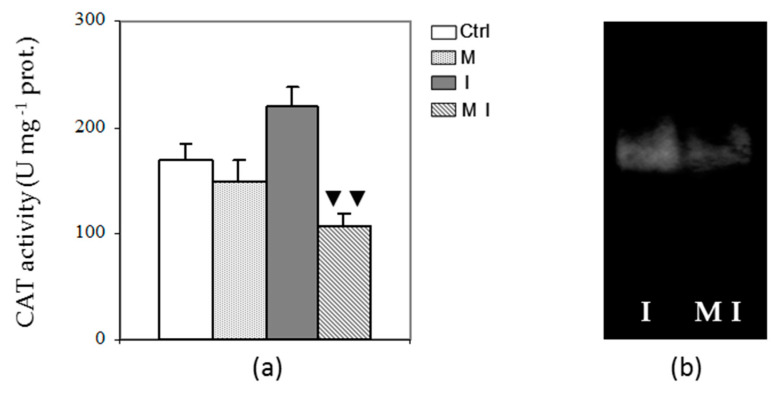
Spectrophotometric activity (**a**) and electrophoretic profile (**b**) in the cytosolic fraction of catalase (CAT) in artichoke control plants (Ctrl), in mycorrhizal plants (M), in plants inoculated with *Verticillium dahliae* (I), and in mycorrhizal plants inoculated with *V. dahliae* (MI); 1 U = 1 nmol H_2_O_2_ dismutated min^−1^; prot. = proteins. The results are given as the mean values of at least five experiments ± SD; ▼▼ indicates values significantly different from the artichoke inoculated with *V. dahliae* (I) by the Student’s *t* test with *p* < 0.01.

Catalase activity was also analyzed aiming to investigate the ability of mycorrhizal plants to detoxify hydrogen peroxide. No significant changes in CAT activity were found between M and Ctrl plants, as shown in Figure 5 (Panel a). In contrast, a high significant (*p* < 0.01) decrease was observed in MI compared to I. This evidence was confirmed by native electrophoresis where I treatments showed a higher band intensity compared to the MI treatment (Figure 5, Panel b).

**Figure 6:**
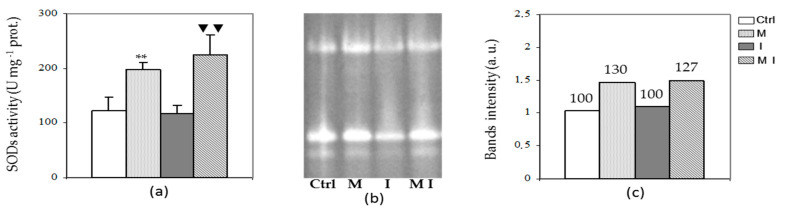
Spectrophotometric analysis (**a**), electrophoretic pattern of Native-PAGE (**b**), and related densitometric analysis (**c**) in the cytosolic fraction of superoxide dismutase (SOD) of artichoke control plants (Ctrl), mycorrhizal plants (M), plants inoculated with *Verticillium dahliae* (I), and in mycorrhizal plants inoculated with *V. dahliae* (MI); 1 U= the amount of enzyme required to inhibit the reduction rate of NBT by 50% at 25 °C; prot. = proteins; a.u. = arbitrary units. The results are given as the mean values of at least five experiments ± SD; * * and ▼▼ indicate values significantly different from the control (Ctrl) and from the artichoke inoculated with *V. dahliae*, respectively, by the Student’s *t* test with *p* < 0.01.

Regarding SOD analysis, the spectrophotometric assay (Figure 6, Panel a), the electrophoretic pattern (Figure 6, Panel b), and the densitometric analysis of the band intensity of Native-PAGE (Figure 6, Panel c), showed a significant increase in its activity in both mycorrhizal plants (M and MI), as compared to control and I plants, respectively. This increase is not due to new additional bands, as three isoforms in all samples have been observed.

### 3.4. H_2_O_2_ Content and Lipid Peroxidation Assay

**Figure 7:**
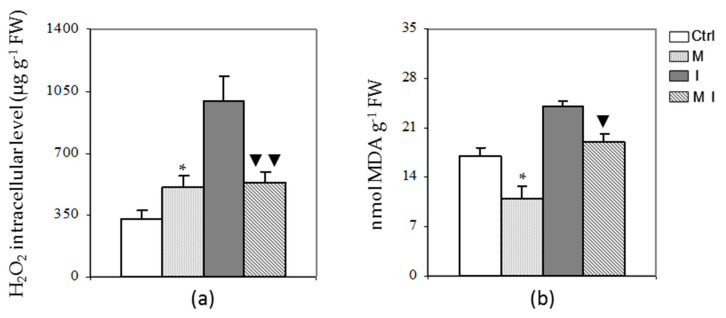
(**a**) Intracellular H_2_O_2_ content in artichoke control plants (Ctrl), in mycorrhizal plants (M), in plants inoculated with *Verticillium dahliae* (I), and in mycorrhizal plants inoculated with *V. dahliae* (MI). The results are given as the mean values of at least five experiments ± SD; * indicates values significantly different from the control (Ctrl) by the Student’s *t* test with *p* < 0.05; ▼▼ indicates values significantly different from the artichoke inoculated with *V. dahliae* (I) by the Student’s *t* test with *p* < 0.01; (**b**) Lipid peroxidation level in artichoke control plants (Ctrl), in mycorrhizal plants (M), in plants inoculated with *V. dahliae* (I), and in mycorrhizal plants inoculated with *V. dahliae* (MI). The results are given as the mean values of at least five experiments ± SD; * and ▼ indicate values significantly different from the control (Ctrl) and from the artichoke inoculated with *V. dahliae* (I), respectively, by the Student’s *t* test with *p* < 0.05.

The effect of mycorrhizal inoculation on ROS accumulation and preservation of membrane structure from oxidative damages after biotic interaction was evaluated by the estimation of H_2_O_2_ content and level of lipid peroxidation. The H_2_O_2_ content was significantly decreased (*p* < 0.01) in MI treatment with respect to the plants inoculated only with the pathogen (Figure 7, Panel a). Similarly, the mycorrhizal plants inoculated or not with the pathogen showed a decreased level of lipid peroxidation as compared to the inoculated and the control plants, respectively (Figure 7, Panel b).

## 4. Discussion

Ensuring stable crop yields and quality while simultaneously guarding human health and the environment is a current challenge facing the farming and research communities. In recent years, the inoculation of plants with AMF has received increasing attention as an environment-friendly approach for improving plant nutrition by increasing nutrients and water availability, nutraceutical values by inducing changes in secondary metabolism, and plant tolerance to biotic and abiotic stress by selecting resistant cultivars and enhancing the activity of antioxidant enzymes [51,52,53,54,55,56]. According to this view, the present study evaluated the effectiveness of the AMF *G. viscosum* as a biocontrol agent against the soil-borne pathogen *V. dahliae* by investigating the antioxidant responses and the effects on ROS metabolism in artichokes. There is sufficient evidence in the literature to confirm that the effect of AMF varies with respect to the host plant and the fungal species [27,57,58,59,60]. Based on previous results where *G. viscosum* showed a better affinity with artichoke plantlets in terms of plant growth and physiological activities, compared with *G. intraradices* [61], we selected the former AMF in this study. Our results showed that inoculation with *G. viscosum* significantly improved productivity and ameliorated the disease severity in most of the AMF-treated plants. This is consistent with previous observations showing a beneficial effect on productivity and disease severity in cotton plants affected by Verticillium wilt inoculated with *Rhizophagus irregularis* [62], in wheat plants infected with *Fusarium pseudograminearum* and colonized by *Rhizophagus intraradices* [56], and in potato contaminated by the pathogen *Fusarium sambucinum* and inoculated with the AMF *Glomus irregular* [63]. Although *G. viscosum* had a significant effect on reducing symptoms’ development on leaves, the inoculation still caused slight stem browning in most of the AMF-treated plants, confirming previous findings where several AMF treatments showed differences in efficiency towards reducing disease severity [57,64,65]. 

The findings of the present study highlighted that the mycorrhization process modulated the activity of the ascorbate–glutathione cycle enzymes, including APX, MDHAR, and GR, and the resulting levels of ASC and GSH. In particular, increased levels of GSH and ASC, along with enhanced activity of APX, GR, and MDHAR, were observed in mycorrhizal artichoke plants compared with non-mycorrhizal controls, while the activity of DHAR, as well as the DHA and GSSG contents, remained unchanged. The effects of AMF on the modulation of ASC-GSH cycle enzymes and antioxidative metabolism have been scarcely studied [66]. Moreover, most of the studies focused on a narrow range of fungi, such as *Trichoderma harzianum* [67], *R. intraradices* [56,68], *Glomus* spp. [69], and the AMF Rhizolive consortium [37]. Previous studies have established a correlation between the level of GSH and resistance to different biotic challenges, including plant viruses, bacteria, and fungi [66,70,71]. Furthermore, GSH represents a key metabolite in the cellular redox buffering system, protecting proteins from irreversible modifications that can be induced by oxidation, through the S-glutathionylation, a post-transcriptional protein modification, which consists in the formation of a stable mixed disulfide between GSH and a protein thiol [72,73]. In this study, the increase in GSH content, observed in both mycorrhizal treatments (M and MI), could be correlated to the higher GR enzyme activity that regenerates the GSH from GSSG, using NADPH as electron donor [74]. Due to the GSH increase, the GSH/GSSG ratio shifts toward the reduced form. Furthermore, the higher availability of NADP+ allows for accepting electrons from photosynthetic electron transport, mitigating the reduction of molecular oxygen to superoxide anion [75]. Moreover, we hypothesize that the increased levels of GSH, ASC, and GSH-dependent enzymes were related with increased mineral elements content (N, P, K, Mg, Fe, Ca, etc.), as demonstrated in previous studies where the higher activity of several antioxidant enzymes was often associated with mycorrhiza-induced increases in biomass and P or N contents [76]. On the other hand, a rise in GSH content is correlated to a higher rate of assimilation of nitrogen and sulfur [77], which are elements of the chemical structure of glutathione.

The increased content of ASC observed in MI plants compared to I, along with the significant increase in MDHAR, corroborates the key role played by MDHAR in the regeneration of ASC from MDHA for ROS scavenging. Moreover, the regeneration of ASC from its oxidative state prevents the intracellular accumulation of DHA that, at high concentrations, has been proved to be toxic for cell metabolisms [78] and to inhibit the activity of enzymes regulated by the thioredoxin–thioredoxin system [79,80]. Our results suggest that MDHAR showed a higher specific activity than DHAR, as reported in previous studies [81,82,83].

The remarkable increase in H_2_O_2_ and MDA in I plants compared with the other treatments was an indicator of oxidative stress caused by pathogen attack. Conversely, in the MI plants, we observed a strong decrease in the H_2_O_2_ level that could be explained by the significant increase in the APX enzyme activity of mycorrhizal plants (MI and I) compared to the other treatments. Similarly, decreased MDA concentrations in leaves of AMF, although at a lesser extent, have been observed. These findings are consistent with published results reporting the antioxidant responses in *Digitaria eriantha* plants inoculated with the AMF *R. irregularis* and subjected to drought, cold, or salinity [51] and the effect of AMF on leaf water potential, solute accumulation, and oxidative stress in soybean plants subjected to drought stress [84]. Furthermore, our findings showed a higher level of H_2_O_2_ in mycorrhizal plants compared to the non-mycorrhizal plants that is consistent with previous results showing that, in the early stages, the establishment of mycorrhizal symbiosis leads to an increase in ROS content followed by an enhanced defense response of the antioxidant system [26,37,62,85,86]. Accordingly, our results showed a significant stimulation of SOD and APX activity in AMF compared to non-AMF samples, indicating lower oxidative damage in the colonized plants. Conversely, the CAT showed a negative response to AMF, while its activity increased in plants inoculated with pathogen. CAT and APX activities are both involved in the scavenging of H_2_O_2_. Although mycorrhizal colonization has been associated with higher antioxidant enzyme activities, the response of the individual enzymes varies with respect to the host plant and the fungal species [58,60]. Moreover, previous studies showed that APX has a much higher affinity for H_2_O_2_ than CAT [87,88,89,90], while high concentrations of H_2_O_2_ induced the expression of genes involved in the synthesis of catalase gene to higher levels, and in less time, than lower H_2_O_2_ concentrations [91]. Furthermore, CAT, APX, and SOD are metalloenzymes depending on micronutrients’ availability, so their activities may be related to the acquisition of Fe, Cu, N, P, and Mn in the plants [58,76]. The activity of SOD, APX, and CAT was also analyzed by using electrophoretic systems. Our results showed the presence of three constitutive isoforms in SOD activity in all samples with an enhanced activity in both mycorrhizal plants, and no induction of new isoforms was detected in plants inoculated with the pathogen (I) or in the mycorrhizal plants. Similarly, three SOD isoforms were found in non-mycorrhizal plants of pepper roots affected by Verticillium wilt, although the colonization with the AMF *Glomus deserticola* induced two new isoforms with similar mobility [85]. 

Overall, our results demonstrated the protective role of the AMF *G. viscosum* on artichoke plants affected by Verticillium wilt through reducing disease severity and enhancing antioxidant systems and activity of investigated antioxidant enzymes. The results of this study suggest that: (1) *G. viscosum* enhances disease tolerance in artichokes; (2) the ascorbate-glutathione cycle plays a key role in maintaining redox balance and avoiding oxidative damage in contaminated artichoke plants inoculated with *G. viscosum*; (3) *G. viscosum* increases the activity of some antioxidant enzymes, such as APX and SOD, while it decreases the activity of some others (CAT), confirming that the efficiency of AMF is related to fungal and/or plant species, soil nutrient availability, and environmental factors. All those data can lend support to the applications of AM *G. viscosum* as a cost-effective and environment-friendly strategy for reducing or alleviating *V. dahliae* effects in artichoke plants.

## Figures and Tables

**Table 1:** Effectiveness of mycorrhizal fungus *G. viscosum* in protecting globe artichoke against Verticillium wilt. For each parameter, different letters (a,b,c) within the same column indicate that the means are significantly different at *p* ≤ 0.05 according to Duncan test. The experiments were repeated three times with three replicates. Ctrl, non-mycorrhizal plants; I, non-mycorrhizal plants inoculated with *V. dahliae*; M, mycorrhizal plants; MI, mycorrhizal plants inoculated with *V. dahlia*.

Treatment	Disease Severity		Flower Heads for Plants
	Foliar Tissue	Vascular System	
Ctrl	0.0 c	0.0 b	13
I	3.9 a	3.0 a	4
M	0.0 c	0.0 b	16
MI	1.6 b	2.7 a	9
